# A magnetic resonance imaging and gut flora-based study of intake desire in overweight/obese type 2 diabetes mellitus patients

**DOI:** 10.3389/fnins.2025.1612722

**Published:** 2025-06-18

**Authors:** Mingxuan Gao, Liya Gong, Yanmei Zeng, Dongling Li, Junyan Wen, Ying Guo, Zhujia Li, Jingwen Luo, Chunling Chen, Ge Wen

**Affiliations:** ^1^Department of Medical Imaging Center, Nanfang Hospital of Southern Medical University, Guangzhou, China; ^2^Department of Endocrinology, Nanfang Hospital of Southern Medical University, Guangzhou, China

**Keywords:** type 2 diabetes mellitus, magnetic resonance imaging, gut flora, neuroimaging, appetite

## Abstract

**Background:**

Abnormal regulation of food intake in overweight/obese type 2 diabetes mellitus (T2DM) patients is an important cause of energy intake imbalance, and studies have demonstrated that the “gut-brain axis” is involved in a wide range of metabolic regulation through neural and endocrine processes, which has become a key breakthrough in revealing the abnormalities of food intake behaviors in T2DM patients.

**Objective:**

Exploring the mechanism of action of abnormal regulation of ingestive desire in overweight/obese T2DM patients by integrating multimodal MRI techniques with microbiological analysis based on the gut-brain axis.

**Methods:**

Thirty-one patients with overweight/obese type 2 diabetes mellitus (Group A), 17 patients with simple obesity with abnormal glucose metabolism (Group B), and 14 patients with simple obesity with normal glucose metabolism (Group C) were recruited, and clinical data, MRI, and stool specimens were collected to analyze the correlation between the imaging indicators and the intestinal flora, and clinical data.

**Results:**

Compared with Group C, the abundance of *Prevotella* and *Bifidobacterium* in Group A and Group B was significantly reduced, while the abundance of *Bacteroides*, *Fusobacterium*, and *Phascolarctobacterium* was significantly increased. Meanwhile, in Group A and Group B, and Bifidobacterium were negatively correlated with HbA1c, thirst score, and FC values of the left supraoccipital gyrus and bilateral thalamus in the population with abnormal glucose metabolism; Bacteroides were negatively correlated with ALFF values of the right inferior frontal gyrus capitellum, and positively correlated with FC values of the left supraoccipital gyrus and bilateral thalamus, and so on.

**Conclusion:**

Abnormal desire to ingest is the result of functional changes in brain regions, dysregulation of flora metabolism and neuroimmune interactions, providing a theoretical basis for clinical diagnosis and treatment targeting the gut-brain axis.

## 1 Introduction

In recent years, with the rapid development and advancement of neuroimaging technology, which provides brand new perspectives for in-depth understanding of the neural mechanisms of ingestive behaviors, multimodal magnetic resonance imaging (MRI), including structural magnetic resonance imaging (sMRI), resting-state functional magnetic resonance imaging (rs-fMRI), and diffusion tensor imaging (DTI), has provided a powerful tool to study the neural mechanisms of the gut-brain axis. SMRI can reveal changes in brain structure, such as changes in gray matter volume and cortical surface area; DTI can assess the microstructural features of white matter fibers; and rs-fMRI can detect abnormalities in functional brain connectivity. The combined application of these techniques can help to comprehensively analyze the brain structural and functional changes in overweight/obese T2DM patients and the relationship with the desire to ingest. Previous studies have revealed that the regulation of the desire to eat involves complex brain network interactions, including key brain regions in the hypothalamus (energy homeostasis regulation), striatum (reward processing), and insula (endosensory processing). In healthy individuals, food cues activate the reward system and trigger feeding motivation, whereas obese individuals often show high responsiveness of reward circuits and inefficiency of inhibitory networks, resulting in a “tendency to overeat” (Brüning and Fenselau, 2023). However, it is unclear whether the mechanisms regulating food intake in overweight/obese T2DM patients differ from those in obese individuals. Diabetes-specific metabolic disturbances (e.g., chronic hyperglycemia, insulin resistance) may affect the function of the central nervous system through the blood–brain barrier, further altering the activity patterns of feeding-related brain regions. For example, insulin not only regulates blood glucose in the periphery, but also acts on hypothalamic neurons to suppress appetite, whereas decreased brain insulin sensitivity in patients with T2DM may diminish this regulatory role and exacerbate uncontrolled ingestion (Abel et al., 2024).

Gut microbiota, as the “second genome,” is deeply involved in the pathological processes of disorders of glucolipid metabolism through metabolites, immune regulation and neural signaling pathways (Sender et al., 2016). It has been found that the gut flora metabolite pantothenate inhibits glucose preference through activation of the GLP-1/FGF21 signaling axis, revealing the central role of gut-liver-brain in metabolic diseases (Zhang et al., 2025). Gut microbes also influence appetite regulation centers in the brain by producing metabolites (e.g., short-chain fatty acids, bile acids) and modulating the secretion of gut hormones (e.g., gastric hunger hormone, GLP-1) (Buhmann et al., 2014). Meanwhile, the vagus nerve, an important conduction pathway of the gut-brain axis, has been found to regulate intestinal absorptive functions (e.g., fat metabolism) in direct association with CNS activity, further supporting the multidimensional influence of the brain and gut in the regulation of ingestion (Lyu et al., 2024).

The gut microbiota plays a central role in the gut-brain axis, and in recent years, microbiological studies have revealed complex interactions between gut microbes and host metabolism, immunity, and neural function. For example, specific probiotic interventions can improve the network of neuronal-glial cell interactions in the brain by modulating intestinal inflammation, metabolite secretion, or vagal signaling, which in turn affects ingestive behavior (Buhmann et al., 2014). In addition, the composition of the gut microbiota is closely related to the metabolic profile of obese and T2DM patients, revealing that microbiological studies may provide new biomarkers and targets for intervention in the diagnosis and treatment of the disease.

However, existing studies have mostly focused on single mechanisms (e.g., microbiome or neuroimaging) and lacked the joint analysis of multimodal neural characterization of the desire to eat and gut microbiome in overweight/obese T2DM patients. In addition, although techniques such as fMRI have been initially applied to the study of neurological function in diabetic encephalopathy, their precise characterization of ingestion-related brain regions (e.g., reward system, hypothalamus, and insular cortex) is still deficient. Therefore, in this study, by integrating multimodal MRI techniques (e.g., rs-fMRI, DTI, etc.) to characterize the brain structure and function of overweight/obese T2DM patients, and combining with microbiological analyses to deeply explore the role of the gut-brain axis in the regulation of ingestive desire, which can help to further understand the pathogenesis of obesity and T2DM, and may also provide the development of a novel treatment strategy based on the gut-brain axis. It may also provide a theoretical basis for the development of novel therapeutic strategies based on the gut-brain axis.

## 2 Materials and methods

### 2.1 Participants

This study was a cross-sectional design with three study groups: overweight/obese T2DM patients group (Group A), simple obesity with abnormal glucose metabolism patients group (Group B), and simple obesity with normal glucose metabolism group (Group C), and the three groups of subjects were matched in terms of age, gender, and education level. Thirty-eight patients with overweight/obese type 2 diabetes mellitus, 22 patients with simple obesity with abnormal glucose metabolism, and 22 patients with simple obesity with normal glucose metabolism who attended the outpatient clinic of the Department of Endocrinology of Zengcheng Branch of the Southern Medical University during the period of September 2023 to September 2024 were recruited, and 19 patients with excessive head movement, artifacts and incomplete data in the images, and 1 patient with unsatisfactory quality test of the fecal sample were excluded. Finally, 31 patients with overweight/obese type 2 diabetes mellitus (Group A), 17 patients with simple obesity with abnormal glucose metabolism (Group B), and 14 patients with simple obesity with normal glucose metabolism (Group C) were included in this study. All participants participated in this study after having sufficient judgment, understanding informed consent and signing a written informed consent form, and all studies were conducted in accordance with relevant guidelines and regulations.

Inclusion criteria for the overweight/obese T2DM group (Group A): (1) age 18–65 years; (2) patients with type 2 diabetes mellitus diagnosed by endocrinologists according to the WHO Diagnostic and Classification Criteria for Diabetes Mellitus (1999), which are as follows: a. diabetic symptoms (polydipsia, polyuria, and abnormal weight loss), and random (at any time after a meal) plasma glucose ≥11.1 mmol/L (200 mg/dL); b. or fasting (fasting for at least 8 h) plasma glucose ≥ 7.0 mmol/L (126 mg/dL); c. or 2h OGTT plasma glucose ≥ 11.1 mmol/L (200 mg/dL); (3) receiving conventional hypoglycemic medications during the illness; (3) right-handed; and (4) signing an informed consent form. Inclusion criteria for the simple obesity with abnormal glucose metabolism group (Group B): (1) age 18–65 years old; (2) 6.1 mmol/L ≤ FPG (fasting glucose) < 7.0 mmol/L, and/or 7.8 mmol/L ≤ 2 h OGTT ≤ 11.1 mmol/L; (3) subjects were dextrorotatory; and (4) signing of informed consent. Inclusion criteria for the group with normal glucose metabolism in simple obesity (Group C): (1) aged 18–65 years; (2) fasting blood glucose <6.1 mmol/L and 2 h OGTT <7.8 mmol/L; (3) not meeting the WHO diagnostic criteria for diabetes mellitus; (4) no history of taking any hypoglycemia-related medication, no history of psychiatric disorders, and no history of drug or alcohol abuse; (5) signing an informed consent form. Exclusion criteria for the three groups: (1) with contraindications to MRI scanning; (2) patients with secondary obesity such as hypothyroidism, Cushing’s syndrome, etc.; long-term use of medications affecting body weight; (3) combination of serious organic diseases such as heart, brain, liver, kidney, etc.; (4) combination of heavy mental disorders such as schizophrenia. (5) Taking antidepressants or any drugs that affect the functional activity of the brain.

### 2.2 Clinical data

Demographic information: diagnosis was determined by cross-consultation between two or more senior endocrinologists (associate physicians and above). We collected basic information about all participants, including name, age, sex, educational background, current medical history, family history, disease duration, and key data such as waist circumference, hip circumference, and BMI. A self-administered general data and clinical characteristics collection form was used.

Biochemical data: Fasting blood glucose, glycosylated hemoglobin, liver function, renal function, and four items of blood lipids were collected from the medical record system of Zengcheng Branch of Nanfang Hospital for all subjects, and all blood tests were done in the laboratory of the Department of Laboratory Medicine of Zengcheng Branch of Nanfang Hospital.

100 mm Appetite Visual Analog Scale (VAS) (Flint et al., 2000; Gibbons et al., 2019; Gonzalez-Izundegui et al., 2021; Raben et al., 1995). A visual analog rating scale was designed to include the assessment of five dimensions: appetite, hunger perception, satiety, satisfaction, and thirst. Each question item was equipped with a 10-cm-long line segment underneath, and subjects were required to mark a vertical line on the segment to reflect the degree of their actual feelings in the current moment.

Dutch Eating Behavior Questionnaire (DEBQ) (Cebolla et al., 2014; Kantonen et al., 2021; Malesza and Kaczmarek, 2021; Van Strien et al., 1986; van Strien et al., 2009; van Strien et al., 2012) assesses the type of eating behavior of the patient, classified as emotional eating, external eating and restrictive eating, and is used to measure different behavioral characteristics. The mean score of the restrictive eating dimension was named “restrictive score,” while the mean scores of the emotional eating dimension and external eating dimension were collectively called “feeding score.”

Control of Eating Questionnaire (COEQ) (Dalton et al., 2015; Hill et al., 1991). The COEQ consists of 21 items divided into 6 sections, which subjects are asked to complete based on how they felt during the past 7 days. A 100 mm visual analog rating scale (VAS) was used to assess the 20 items. The above questionnaires were completed by the same clinician.

### 2.3 MRI data acquisition

MRI scans of all subjects in this study were done in the imaging department of Southern Hospital Zengcheng Branch. MRI examinations were performed on all subjects, prior to which the MRI equipment was calibrated for data stability. During the scanning process, the subjects were placed supine on the examination bed, and the head position was stabilized by using head restraint straps and foam pads to minimize the interference of head movements on the MRI signal acquisition. Prior to the start of the examination, subjects were instructed to keep their eyes closed and relaxed throughout the examination, to avoid falling asleep, to maintain consciousness, and to minimize unnecessary mental activity. The scanning equipment used was a GE 3.0 T MRI scanner (SIGNA Architect, GE Medical System, the United States) with a 48-channel standard head coil with the following parameters.

The structural information of 3D-T1-weighted images (3D-T1WI) was acquired using a brain volume scanning sequence, which was realized by a 3D magnetization-prepared rapid gradient echo sequence (MP-RAGE) with the following parameters: Flip Angle (FA) = 15°, Inversion Time (TI) = 1,000 ms, Slice Thickness (SL) = 1 mm, Space Between Slice = 1 mm, Repetition Time (TR) = 2,338.3 ms, Echo Time (TE) = 3.112 ms, Number of Slices = 392, FOV = 256 mm × 256 mm, Matrix = 5.112 ms, FOV = 256 mm × 256 mm. 256 mm, matrix = 512 × 512, bandwidth (Bandwidth) = 122 Hz/Px, and voxel size of 0.5 mm × 0.5 mm × 0.5 mm.

The BOLD imaging technique used the method of Spin Echo-Echo Plane Imaging (SE-EPI) in a spin-echo sequence with TR/TE = 2000/30 ms, FA = 90°, layer thickness = 3 mm, layer spacing = 4 mm, space between slice = 1 mm, FOV = 220 × 220 mm, matrix = 64 × 64, 32-layer axial image covering the whole brain range, 180 volumes of data were acquired continuously by means of axial scanning.

DTI sequence: TR/TE = 10,000/70 ms, FA = 90°, FOV = 256 × 256 mm, matrix = 256 × 256, 1 acquisition of the signal, layer thickness = 2 mm, number of layers = 70, no interval acquisition, and incorporating parallel acquisition techniques to reduce image deformation, a diffusion-sensitive gradient (b = 1,000s/mm^2^) was applied in 64 non-collinear directions for Data Acquisition.

### 2.4 MRI data analysis

For the preprocessing of 3D-T1-weighted imaging (3D-T1WI) structural data, we used the Freesurfer image analysis software platform,^1^ which is able to achieve cortical and subcortical nuclei precise segmentation of cortex and subcortical nuclei and extraction of relevant quantitative parameters. The preprocessing process started with the conversion of all raw high-resolution 3D-T1WI data from dcm format to nii format using the dcm2nii tool.^2^ Subsequently, these “nii” format data were imported into a Linux system for further processing via Freesurfer software. In this process, the data were converted to mgh format and accompanied by the generation of 8 key folders including “label,” “mri,” “stats,” etc. The default script of Freesurfer was used to convert the data from “dcm” to “nii” format. Using Freesurfer’s default script “recon-all,” we performed cortical segmentation and specific computation of brain parameters.

Preprocessing of BOLD-fMRI data was achieved through the DPARSF component of the DPABI toolbox^3^ and included conversion from the data format, removal of the first 10 unstable time points, temporal layer correction, cephalomotion correction, cranial stripping, spatial normalization based on the DARTEL algorithm, linear drift removal, regression covariate variables (including Friston24 head movement parameters, white matter signal, and cerebrospinal fluid signal). In addition, subjects with excessive head movements were excluded (subjects with head movements greater than 3 mm or 3° in any frame), and at a later stage of the preprocessing process, filtering was taken to limit the frequency range of the analysis, followed by smoothing (Gaussian kernel = 4 × 4 × 4 mm^3^) to reduce noise and improve spatial continuity of the data. The pre-processed BOLD-fMRI data were imported into DPARSF of the DPABI software toolbox using age and gender as covariates, and the resting-state functional MRI metrics ALFF and ReHo were calculated for each subject. According to the previous literature, the thalamus, frontal lobe, and orbital frontal cortex and the clusters of resting-state functional metrics that were altered in the present study were selected as the seed points (Cooper et al., 2017; Reppucci and Petrovich, 2016; Xu et al., 2020; Zhang, 2020), and the data were analyzed via the Marsbar toolbox^4^ to produce the ROIs of the regions of interest, and then the FC maps between the ROIs and the whole brain voxels were calculated by the DPARSF software of the DPABI toolbox. The correlation coefficient r was obtained by calculating the average time series of each region of interest (ROI) and correlating it with the time series of other voxels in the whole brain of the subjects, and in order to improve the normality analysis of the data, the correlation coefficients were transformed from Fisher’s to z to obtain the z-score matrix, and the z-score values were used in the next step of statistical analysis.

Diffusion data and white matter fiber bundle imaging were processed according to a standard procedure using DSI studio software.^5^ Regions with significant BOLD-fMRI differences were selected as regions of interest to explore their structural connectivity. All ROIs were expanded by a voxel into the white matter so that they were in contact with the fibers, and metrics such as mean fractional anisotropy (FA), mean diffusion coefficient value (MD), and fiber length were extracted from all subject fibers by deterministic fiber tracking of fiber bundles from each pair of symmetric ROIs.

### 2.5 Intestinal flora data processing

Fecal samples were collected from all subjects on the day of symptom assessment, and subjects were instructed to take care to lay down the padded paper first when sampling and not to introduce other stray bacterial infections in the environment, to use a special 3 mL fecal specimen collection and preservation tube to take the mid-portion of feces (~500 mg), and to minimize the fecal samples’ exposure to the air, and to submerge the collected samples in the resolving preservation solution, and to gently invert the preservation tube for 10 times, and to sufficiently The samples were mixed well, labeled with sample information, and sent to Guangzhou Kidio Technology Service Co., Ltd. and stored at −80°C before analysis for further high-throughput 16S ribosomal DNA gene sequencing.

### 2.6 Statistical analysis

#### 2.6.1 Clinical data

Demographic and clinical data were analyzed using SPSS27.0 software, comparison of gender and type of eating behavior among the three groups was done using chi-square test, *p* < 0.05 indicated significant difference, demographic information, biochemical indicators and scale scores were analyzed using one-way ANOVA, ANOVA results were varied, *post hoc* test two-by-two comparisons were done using Bonferroni, *p* < 0.05 indicated a statistical difference.

#### 2.6.2 MRI data

For gray matter morphology data, we implemented one-way ANOVA between the three groups, and if the ANOVA results showed significant differences, two-by-two comparisons were performed using Bonferroni post-hoc test, with *p* < 0.05 indicating statistical differences. fMRI data were analyzed with DPABI, and comparisons between the three groups were performed using one-way ANOVA, with age and gender as covariates, and ANOVA Differences in results were compared two by two using Bonferroni *post hoc* test, with *p* < 0.05 indicating statistical differences, and Gaussian Random Field (GRF) multiple comparisons were corrected to correct for single voxel *p* < 0.001, and regions with cluster size *p* < 0.05 were considered to be statistically different brain regions, with two-tailed correction. Deterministic fiber tracking was used to extract white matter DTI eigenvalues, and the data were analyzed by one-way ANOVA, with differences in ANOVA results, and two-by-two comparisons using Bonferroni post-hoc test, with *p* < 0.05 indicating a statistical difference.

#### 2.6.3 Intestinal flora data

The raw sequencing data were quality filtered using FASTP to obtain clean reads, which were subsequently spliced using FLASH (minimum overlap length 12 bp, maximum mismatch rate ≤2%). After secondary filtering to remove low-quality sequences, denoising analysis was performed using the DADA2 plug-in for QIIME2: a sample-specific error model was constructed by machine learning to correct sequencing errors and generate amplicon sequence variants (ASVs), while chimeras were removed using the UCHIME algorithm. Species annotation was accomplished by comparing the SILVA database with a plain Bayesian approach (confidence threshold 0.8–1) for RDP classifiers. Statistical analyses were performed using the Vegan package for the Kruskal-Wallis H-test and Tukey’s HSD test, combined with LEfSe to screen for biomarkers. Circos was utilized to map species abundance rings. The whole process integrated de-redundancy, error correction, chimera filtering and standardized statistical methods to ensure data reliability and analytical reproducibility.

Finally, Spearman’s correlation analysis was used to correlate the associations between gray matter morphology data, fMRI indices, DTI eigenvalues, intestinal flora and clinical symptoms.

## 3 Results

### 3.1 Comparison of clinical data

This study included 31 patients with overweight/obese T2DM mellitus (Group A), 17 patients with simple obesity with abnormal glucose metabolism (Group B), and 14 patients with normal glucose metabolism in simple obesity (Group C), totaling 62 subjects.

The statistics of the clinical data of the three groups of subjects are shown in Table 1. In terms of demographic data, the proportion of males in Group A was significantly higher than that in Groups B and C, and the age was higher than that in Group B. The BMI of Group A was lower than that of Group C. The waist-to-hip ratio of Group A was higher than that of Groups B and C. In terms of biochemical indexes, the FPG, HbA1c, 2 h-PG, and HOMA-IR of Group A were higher than those of Groups B and C, while the HOMA-*β* was lower than those of Groups B and C. Moreover, the appetite score of Group A was higher than that of Group C. All of the above were statistically different (*p* < 0.05), while there was no significant difference in the type of eating behavior among the three groups.

**Table 1:** Statistical table of clinical data of the three groups.

Measures		A (*n* = 31)	B (*n* = 17)	C (*n* = 14)	F/ꭓ^2^	*P*
Sex Male	Male	22 (71.0%)	9 (52.9%)	3 (21.4%)	9.591	0.008^*#^
	Female	9 (29.0%)	8 (47.1%)	11 (78.6%)		
Age		38.94 (8.52)	32.18 (7.72)	34.71 (7.52)	10.712	0.022^*^
BMI (kg/m^2^)		29.21 (3.34)	28.81 (7.88)	33.63 (5.78)	43.825	0.028^#^
WHR		0.97 (0.06)	0.89 (0.06)	0.91 (0.10)	43.730	0.001^*#^
FPG (mmol/L)		8.65 (3.17)	5.33 (0.89)	5.33 (0.50)	16.086	<0.001^*#^
HbA1c (%)		9.44 (2.88)	6.32 (1.32)	5.90 (0.33)	22.737	<0.001^*#^
2 h-PG (mmol/L)		12.65 (4.12)	8.99 (2.40)	8.14 (1.89)	12.794	<0.001^*#^
HOMA-IR		9.90 (5.34)	6.32 (3.29)	5.15 (1.86)	9.478	<0.001^*#^
HOMA-β		121.78 (75.18)	305.85 (157.74)	261.59 (140.31)	14.429	<0.001^*#^
Appetite		3.56(2.97)	3.62 (2.60)	1.40 (1.98)	3.577	0.034^#^

Overweight/obese T2DM patients group (Group A), simple obesity with abnormal glucose metabolism patients group (Group B), and simple obesity with normal glucose metabolism group (Group C). Only statistically significant indicators are listed; sex is shown as the number (percentage in the group), and the rest of the data are shown as mean (standard deviation); “*” indicates the comparison between A group and B group (*p* < 0.05); “#” indicates the comparison between A group and C group (*p* < 0.05).

### 3.2 Comparison of magnetic resonance imaging data

#### 3.2.1 Morphological indicators of gray matter

**Figure 1:**
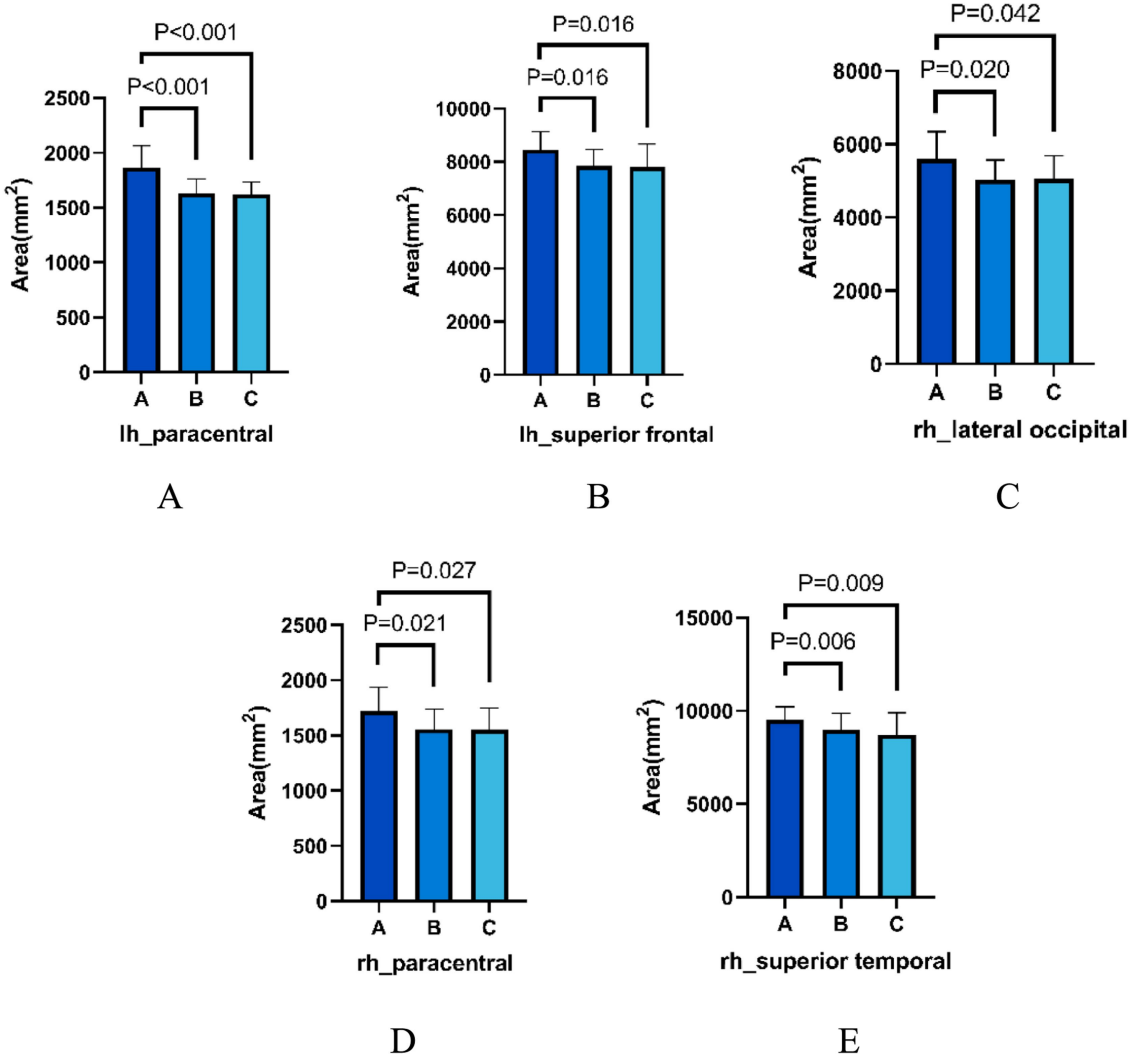
**(A–E)** show, respectively, the area comparison of the cortex of the left paracentral, left superior frontal gyrus, right lateral occipital lobe, right paracentral and right superior temporal gyrus in the three groups.

Brain regions that differed in all three groups in terms of gray matter morphometric indices are shown in Figure 1. Analysis of gray matter morphometric indices of structural images revealed that the cortical areas of the left paracentral lobule, the left superior frontal gyrus, the right lateral occipital lobe, the right paracentral lobule, and the right superior temporal gyrus were significantly greater in group A compared with groups B and C. The results were corrected for Bonferroni (*p* < 0.05).

#### 3.2.2 Resting-state functional magnetic resonance metrics

**Figure 2:**
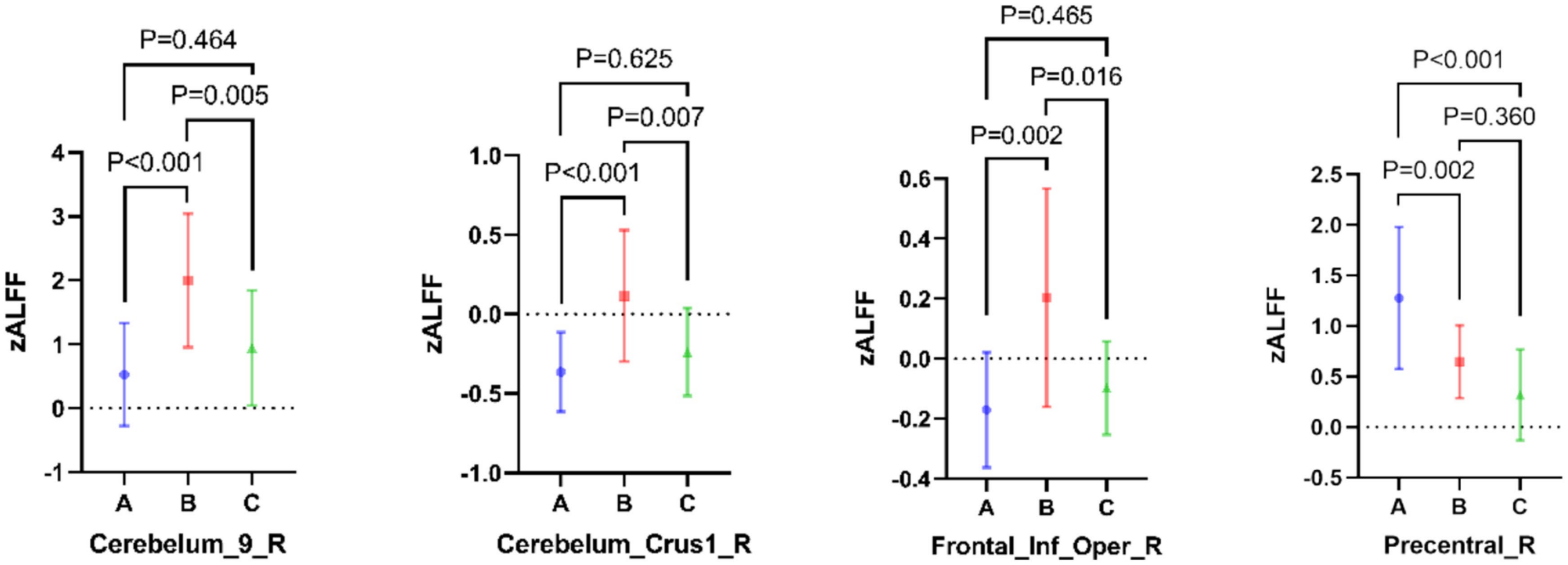
Cluster with differences in amplitude of low-frequency fluctuation (ALFF) among the three groups.

Compared with Groups B and C, Group A had decreased ALFF in localized clumps in the cortex of the right lobule IX of cerebellar hemisphere, right crus I of cerebellar hemisphere, and right inferior frontal gyrus-opercular part. However, Group A had higher ALFF in localized masses in the right precentral gyrus cortex than Groups B and C. Group B had increased ALFF in localized masses in the right lobule IX of cerebellar hemisphere, right crus I of cerebellar hemisphere, right inferior frontal gyrus-opercular part and right precentral gyrus, compared to Group C. See Figure 2 for details (*p* < 0.05, GRF corrected).

#### 3.2.3 Functional brain connectivity

**Figure 3:**
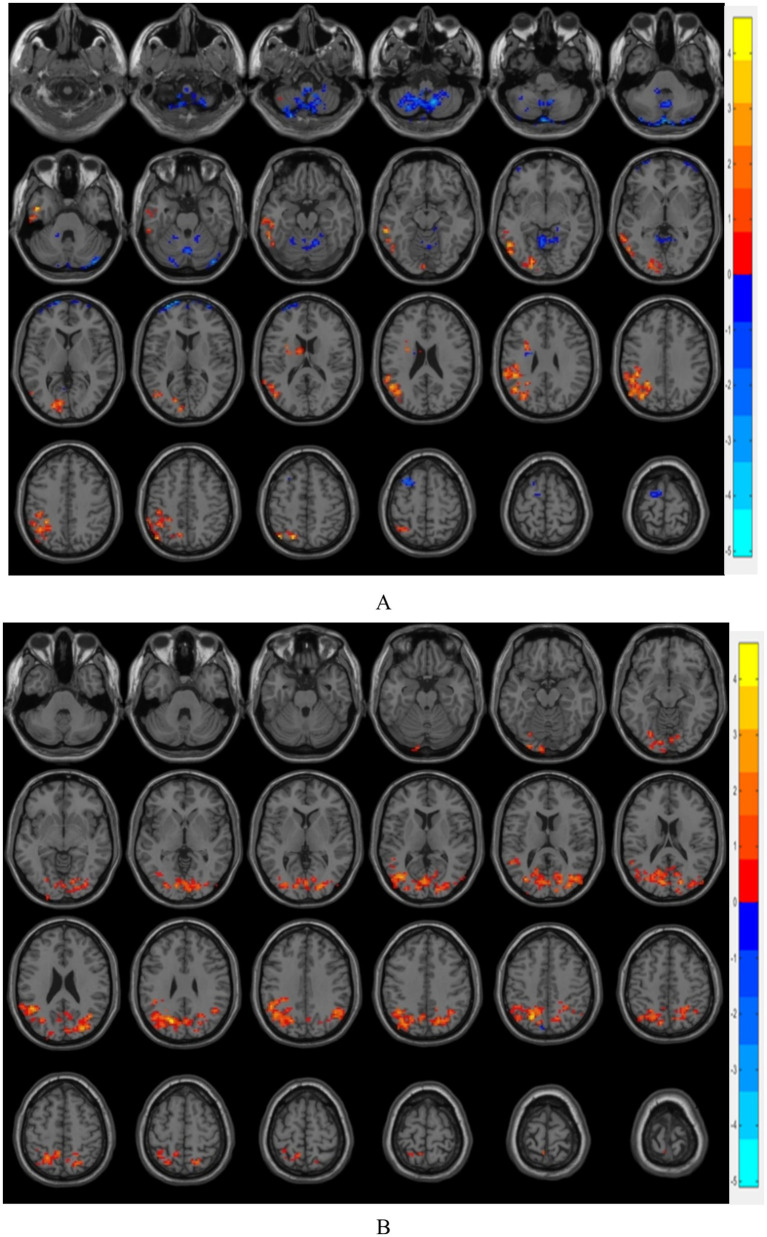
**(A)** shows the brain regions with differences in FC between A group and B group, and **(B)** shows the brain regions with differences in FC between A group and C group (*p* < 0.05, GRF corrected); warm color indicates that the strength of functional connection is increased, and cold color indicates that the strength of functional connection is reduced.

**Figure 4:**
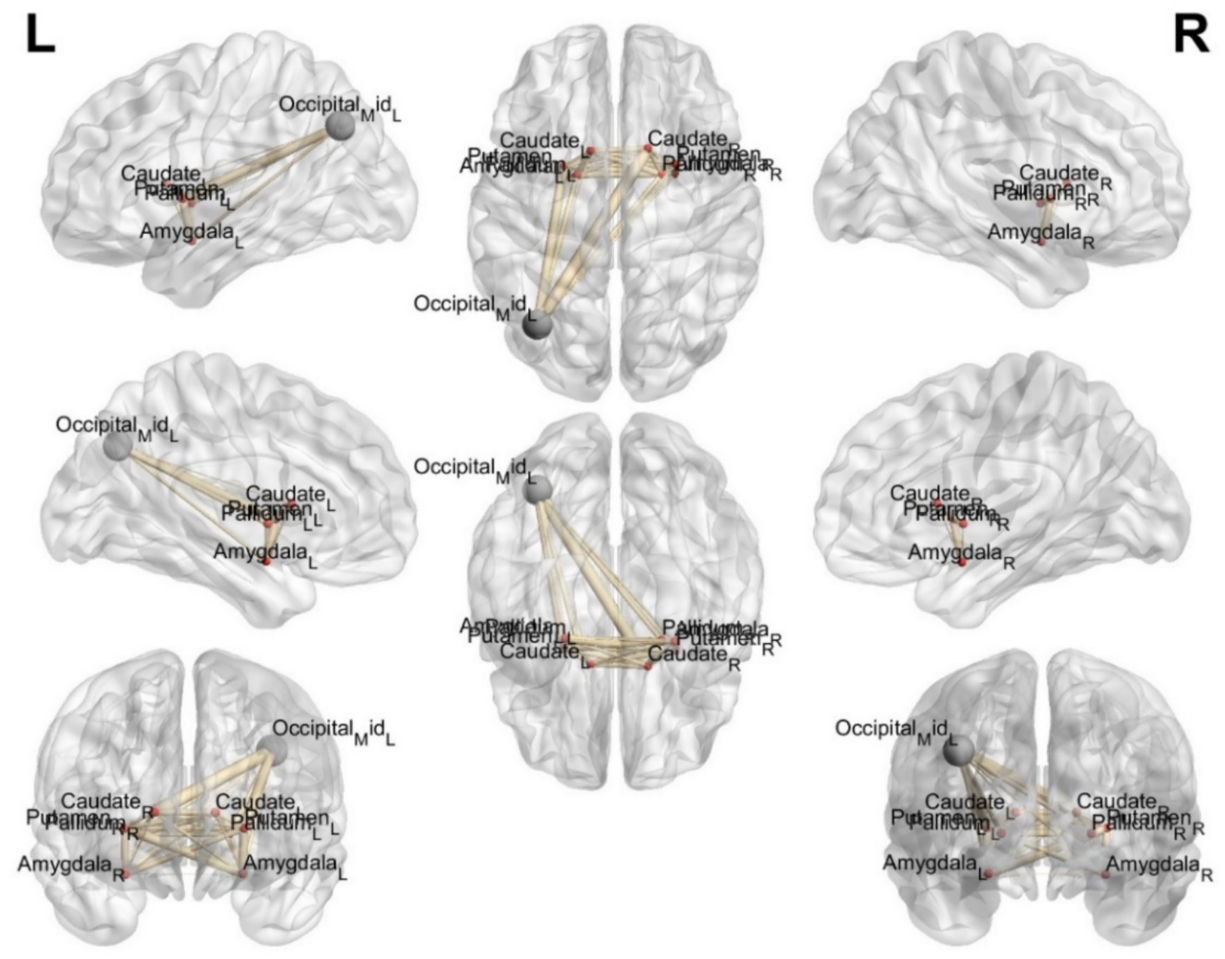
BrainNet software visualization of resting-state functional connectivity strength between ROIs in the region of interest and ROI nodes with differences in FC between the three groups; gray indicates ROI nodes with differences in FC, red indicates ROI nodes in the region of interest.

The ROI of the region of interest and the functional connectivity strength of the localized mass in the left middle occipital gyrus were significantly altered among the three groups, as follows: (1) compared with group B, the functional connectivity strength of the left middle occipital gyrus with bilateral thalamus, the nucleus pulposus, the nucleus accumbens, and the amygdala was enhanced in group A. (2) compared with group C, the functional connectivity strength of the left middle occipital gyrus with bilateral caudate nucleus was enhanced in group A. The above are all statistically different (*p* < 0.05, GRF corrected). See Figures 3, 4 for details.

#### 3.2.4 Diffusion tensor imaging metrics

According to the results of significant differences in resting-state functional indexes of the three groups, the bilateral middle occipital gyrus, thalamus, nucleus pulposus, nucleus accumbens, amygdala, and caudate nucleus were selected as the ROIs of regions of interest, and the deterministic fiber tracking was performed in DSI studio software to obtain the FA value, MD value, AD value, and RD value of each ROI, and the three groups of DTI indexes were subjected to a one-factor ANOVA analysis using the LSD *Post hoc* test was used to compare two by two (*p* < 0.05), see Table 2 for details, the results were as follows: compared with groups B and C, the AD value of the shell nucleus of the right soybean nucleus in group A was decreased; compared with group C, the average length of the white matter fiber bundles, the GFA value, and the AD value of the pale globe of the right soybean nucleus in group A were decreased; compared with group C, the GFA value of the right soybean nucleus of the pale globe in group B was increased, and all of the above were statistically different (*p* < 0.05).

**Table 2:** Comparison of DTI indexes of white matter fiber tracts in three groups.

Measures		A (*n* = 31)	B (*n* = 17)	C (*n* = 14)	*P*
Mean length (mm)	rPAL	41 (3)	45 (5)	43 (7)	0.016^*^
GFA	rPAL	0.081 (0.004)	0.084 (0.003)	0.081 (0.005)	0.034^*^^
AD	rPUT	1.11 (0.02)	1.12 (0.02)	1.12 (0.03)	0.028^*#^
(10^−3^ mm^2^/s)	rPAL	1.13 (0.04)	1.15 (0.03)	1.14 (0.03)	0.040^*^

Overweight/obese T2DM patients group (Group A), simple obesity with abnormal glucose metabolism patients group (Group B), and simple obesity with normal glucose metabolism group (Group C). the data is displayed as the mean (standard deviation), “*” indicates the comparison between A group and B group (*p* < 0.05); “#” indicates the comparison between A group and C group (*p* < 0.05); “^” indicates the B group is *p* < 0.05 compared with the C group.

### 3.3 Comparison of intestinal flora

#### 3.3.1 Alpha diversity analysis

**Figure 5:**
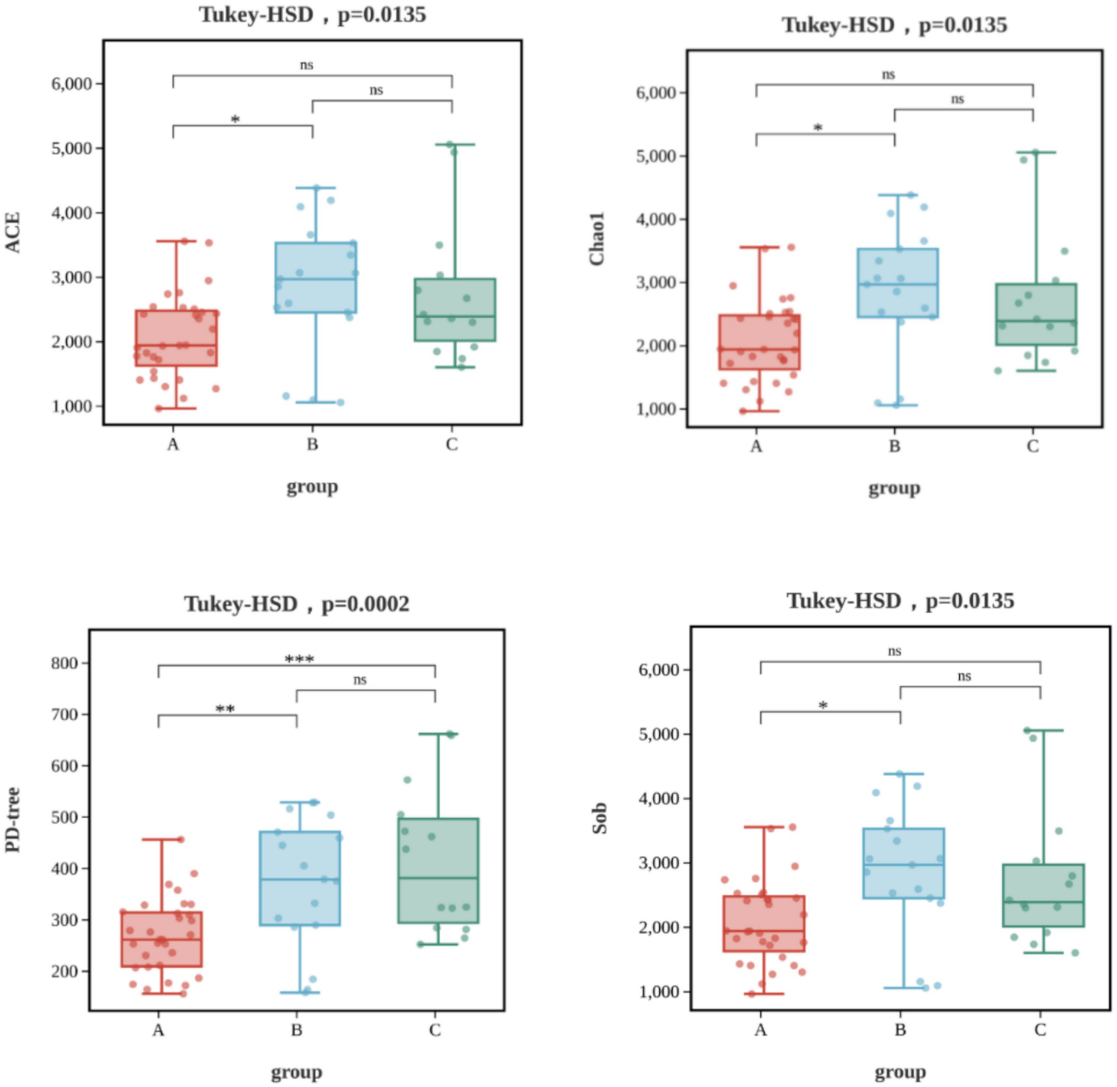
Box diagram of α diversity index among three groups The number of “*” indicates the degree of difference between the two groups, and “ns” indicates that the difference between the two groups is not statistically significant (*p* > 0.05).

The results of this study found that compared to groups B and C, the Chao1, ACE, Sob, and PD-tree indices in group A were decreased and significantly different (*p* < 0.05), and the abundance of intestinal flora in overweight/obese T2DM (group A) population was significantly decreased compared to that of the purely obese population. See Figure 5 for details.

#### 3.3.2 Beta diversity analysis

**Figure 6:**
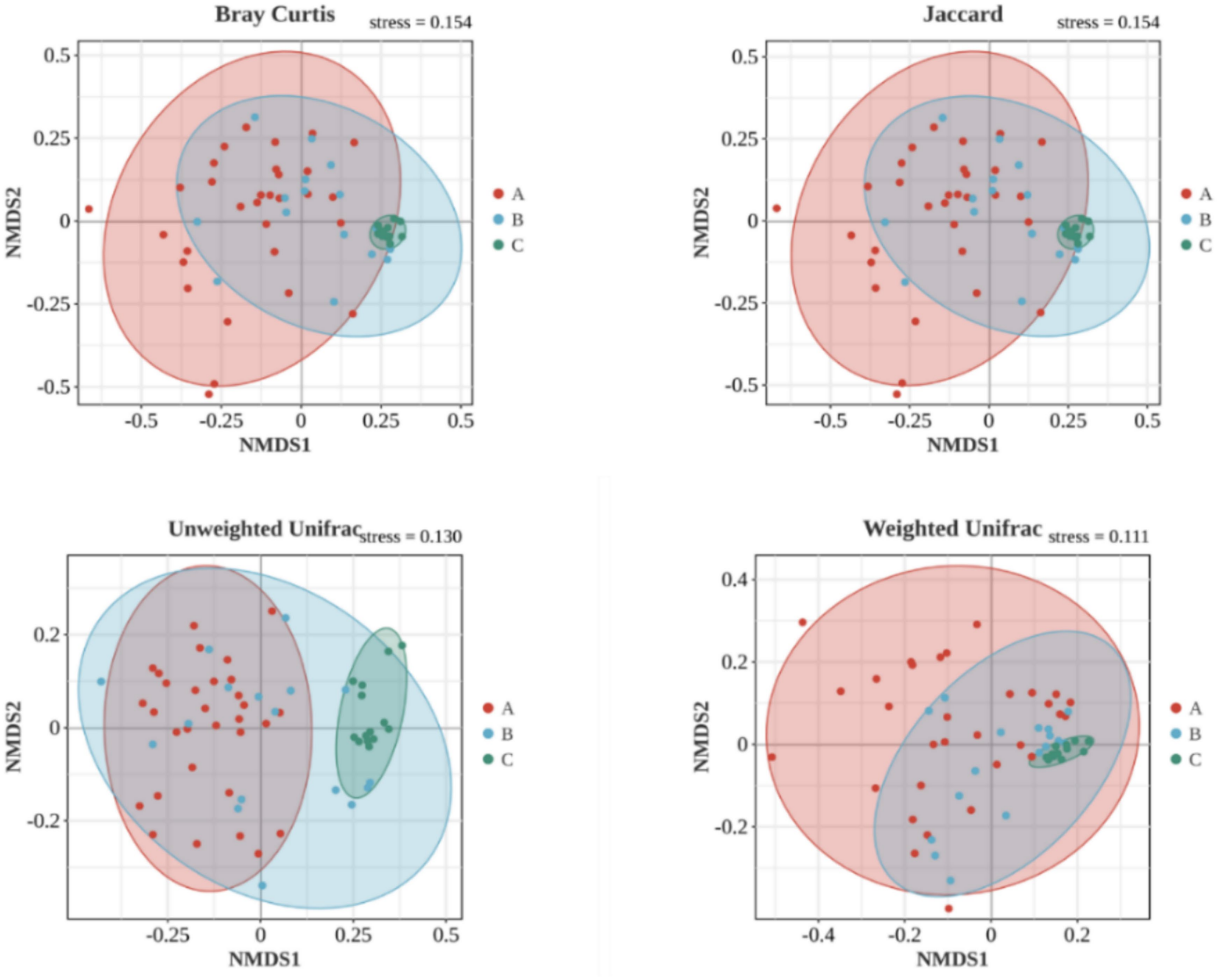
Difference analysis of *β* diversity index of intestinal flora among three groups (based on NMDS).

**Figure 7:**
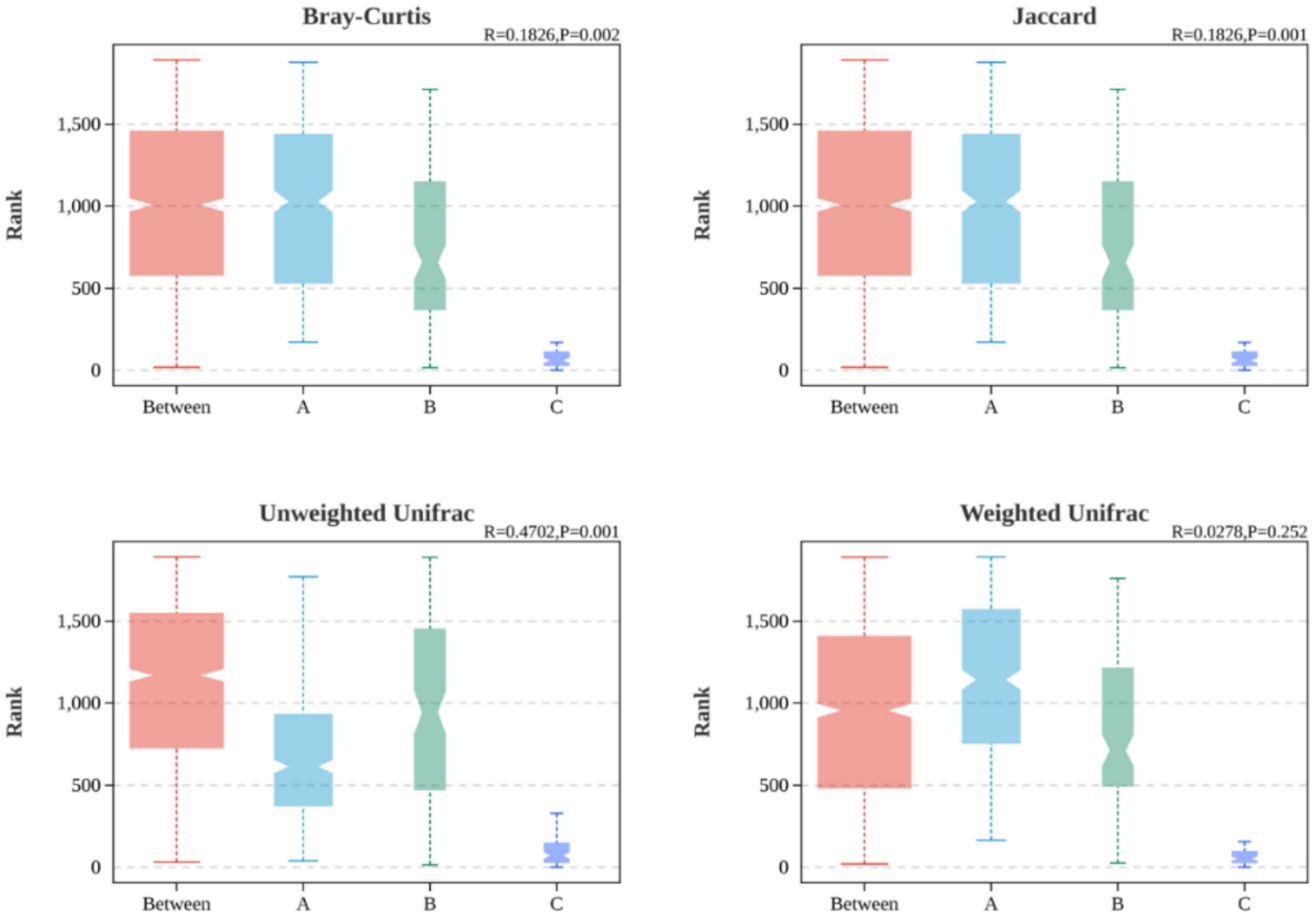
ANOSIM boxplot based on comparison of three groups of *β* indicators.

Based on ANOSIM analysis, the *β* diversity indices of the samples at Bray Curtis, Jaccard and Unweighted Unifrac were significantly different (*p* < 0.05) among the three groups, as shown in Figures 6, 7, indicating that in comparison with the purely obese population with abnormal glucose metabolism and the purely obese population with normal glucose metabolism, there was a significant difference (*p* < 0.05) in the microbial species of the overweight/obese T2DM population in terms of β diversity was significantly different (*p* < 0.05).

#### 3.3.3 Analysis of differential species

**Figure 8:**
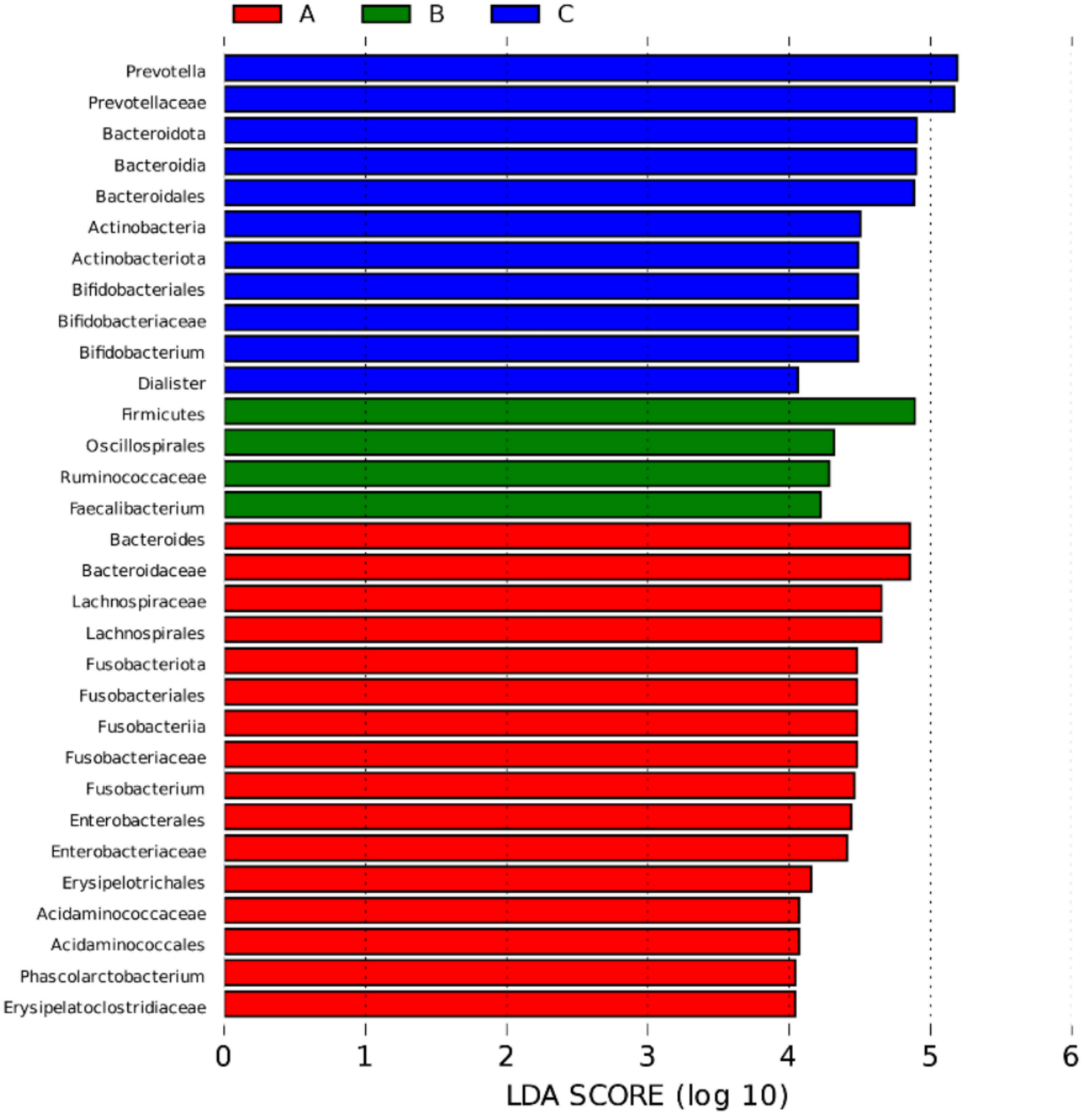
Histogram of LDA value distribution in LEfSe analysis of different species among three groups Red, green, and blue colors indicate species enriched in groups A, B, and C, respectively; the length of the bar graph represents the magnitude of the effect of the species on the between-group differences, and LDA > 2 indicates that the species partitioning between the two groups is statistically different.

**Figure 9:**
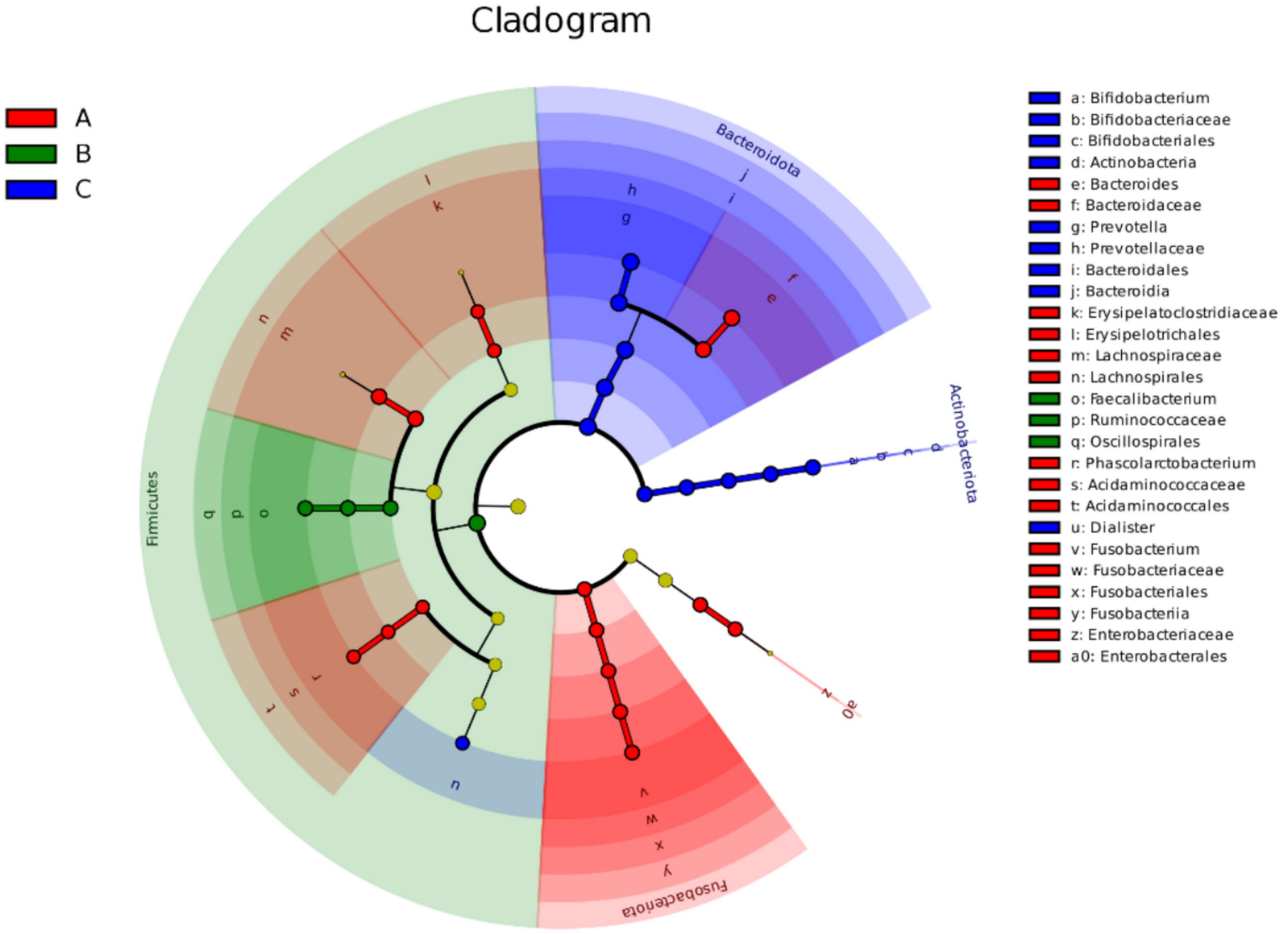
Evolutionary branching diagram of different species among three groups From inside to outside are phylum, order, order, family, genus and species, red, green and blue color indicate different microbial taxa in group A, B and C respectively, yellow color indicates no significant difference between groups, and the size of the circle represents the degree of species classification.

LEfSE analysis of the fecal microbial community composition of the three groups was performed, and Figures 8, 9 shows the characteristic bacteria based on the genus level log LDA threshold of 4. Sixteen species were enriched in group A, mainly enriched by *Bacteroides*, *Lachnospira*, *Fusobacterium*, etc.; four species were enriched in group B, including *Firmicutes*, *Faecalibacterium*, etc.; 11 species were enriched in Group C, including *Prevotella*, *Bacteroides*, etc.

**Figure 10:**
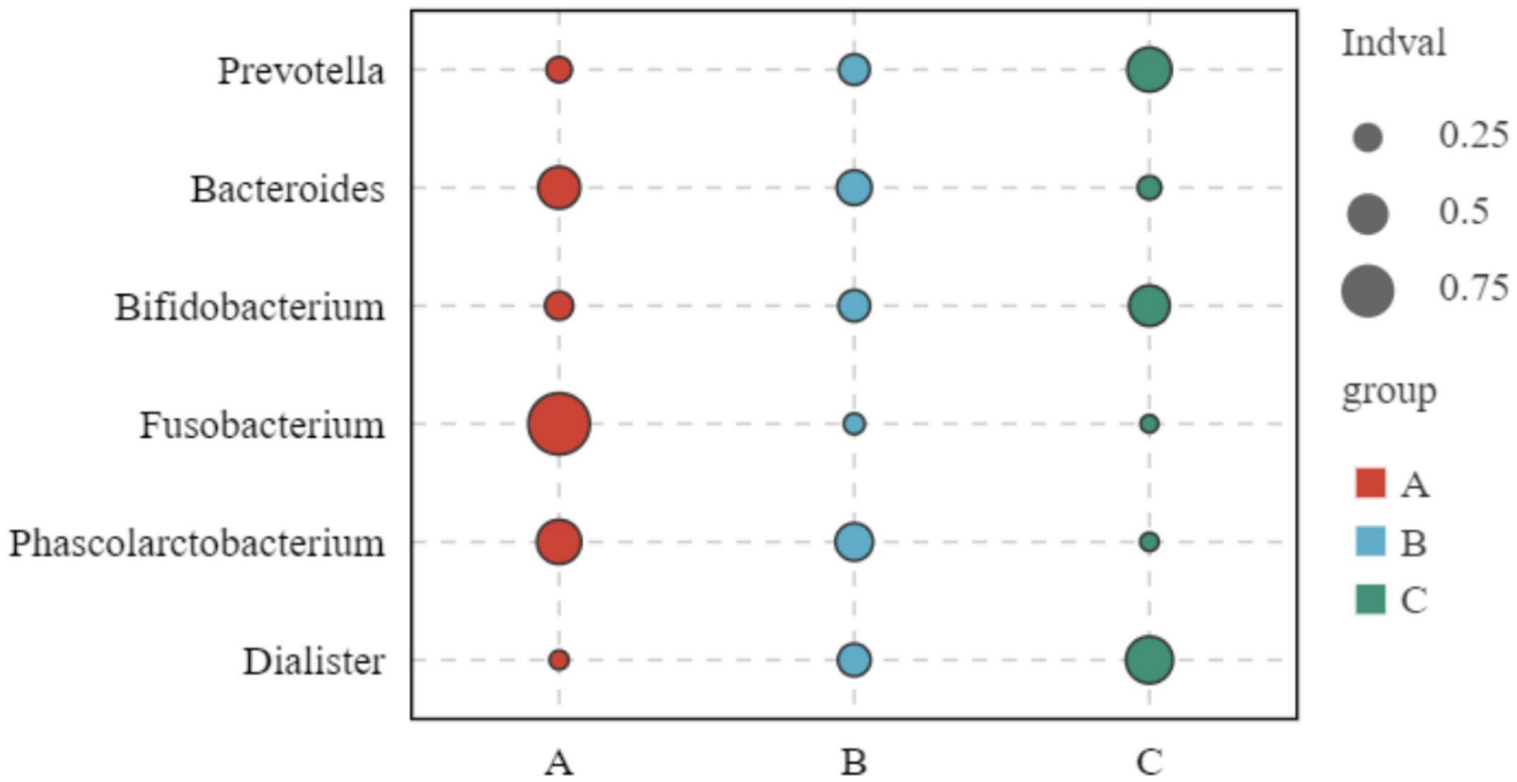
Biomarker bubble chart of three groups at genus level Indval (indicator value): the ratio of specificity to occupancy, used to identify key species in the community; bubble size indicates the indicative size of the species in the grouping, and bubble color indicates the grouping; the vertical axis is the species that differ between the three groups.

The results showed that the relative abundance of *Prevotella* and *Bifidobacterium*, which are the characteristic bacteria of group C, gradually increased among groups A, B and C. The relative abundance of the characteristic bacteria of group A, such as *Bacteroides*, *Fusobacterium* and *Phascolarctobacterium*, gradually decreased among groups A, B and C. All of the above were significantly different (*p* < 0.05), see Figure 10.

### 3.4 Correlation analysis

In groups A and B, the imaging characteristic indexes and clinical data with significant differences among the three groups, and the abundance of intestinal flora with significant differences were selected to do correlation analysis, and the results were shown in Figure 10.

**Figure 11:**
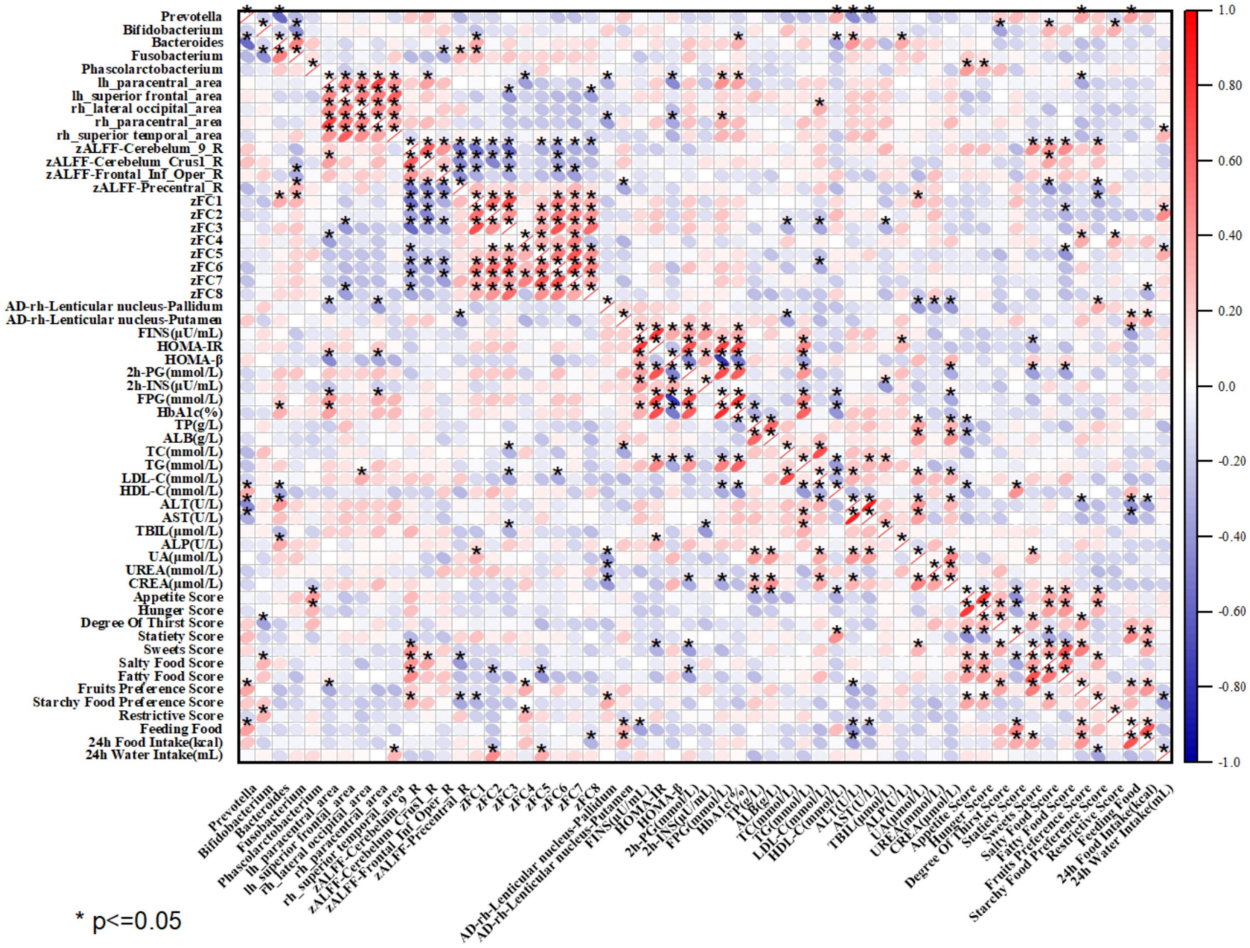
Heatmap of the correlation between imaging indices, intestinal flora and clinical data; positive correlation in red, negative correlation in blue; “*” indicates *p* < 0.05.

*Prevotella* was positively correlated with HDL-C, negatively correlated with ALT, AST, and positively correlated with salty food preference and fruit preference scores; *Bifidobacterium* was negatively correlated with HbA1c, FC values of the left occipital gyrus-bilateral thalamus, the anterior abdominal nucleus and the posterior lateral aspect, and thirst scores, and positively correlated with ALT, restriction score, and feeding score; *Bacteroides* was negatively correlated with HDL-C, ALFF values in the right inferior frontal gyrus capitulum, and positively correlated with ALP, ALFF values in the right inferior frontal gyrus-opercular part, and positively correlated with ALP, ALFF values in the right precentral gyrus, FC values of the left occipital gyrus-bilateral thalamus, the anterior abdominal nucleus and the posterior lateral aspect; and Phascolarctobacterium was positively correlated with appetite and hunger scores and negatively correlated with fruit preference scores (see Figure 11).

## 4 Discussion

The microbiota-gut-brain axis (MGBA) is a complex neural-humoral-immune network system that encompasses the central nervous system, the autonomic nervous system, the neuroendocrine pathway, and the hypothalamus-pituitary–adrenal axis, which realizes bidirectional information communication between the gut and the brain (Zyoud et al., 2019). Studies have revealed that in the pathogenesis of patients with type 2 diabetes mellitus (T2DM), MGBA acts mainly through three core pathways: the neural pathway, the neuro-endocrine pathway, and the neuro-immune pathway (Westfall et al., 2017).

In this study, overweight/obese T2DM patients were found to have increased cortical surface area in brain regions such as bilateral central lobules and left superior frontal gyrus, suggesting that these brain regions associated with somatic movement regulation and cognitive execution may respond to the effects of long-term metabolic disorders on neuroplasticity through compensatory structural expansion (Bernardes et al., 2018). Resting-state functional magnetic resonance further revealed abnormal ALFF values in the cerebellar hemispheres and the right precentral gyrus, suggesting that an imbalance in the functional activity of the cerebellar-cortical loop may be impairing fine regulation of feeding behavior (Xia et al., 2013). The right inferior frontal gyrus, as a key region of prefrontal cortex, is involved in impulse control and executive functions, in which dopamine and GABAergic systems are sensitive to insulin, and insulin resistance reduces GABA synthesis and decreases inhibitory neuronal activity, leading to elevated ALFF. The cerebellum is not only a motor coordination center, but also participates in metabolic regulation and reward processing. Previous studies have shown that chronic hyperglycemia leads to cerebellar microangiopathy and blood–brain barrier damage, which triggers neuroinflammation. Elevated ALFF may be a compensatory response of the cerebellum to try to maintain energetic homeostasis by enhancing local neural activity. Studies have confirmed that increased expression of inflammatory factors in the cerebellum of diabetic mice is associated with neuronal hyperexcitability (Shim et al., 2018). Cerebellar neurons express insulin receptors, and insulin signaling is involved in regulating synaptic plasticity in Purkinje cells. Insulin resistance can lead to impaired glucose uptake in the cerebellum, and neurons require enhanced electrical activity to maintain function, as evidenced by elevated ALFF. Clinical studies have shown that enhanced cerebellar-cortical connectivity in T2DM patients correlates with decreased executive function. The cerebellum interacts with the gastrointestinal through the vagus nerve and is involved in glucose regulation. Gastrointestinal dyskinesia in diabetic patients may activate the cerebellum through visceral afferent signaling, leading to abnormally elevated ALFF (Dai et al., 2024). Other studies have revealed that decreased ALFF in the posterior lobe of the cerebellum in patients with T2DM is negatively correlated with insulin resistance and glycated hemoglobin, suggesting that diminished cerebellar activity may reflect impaired metabolic regulation in hyperglycemic states (Balsters et al., 2013).

The combination of the two can be speculated that increased cortical area may partially counteract the impairment of executive function due to metabolic disturbances by enhancing cognitive control, but the abnormal ALFF suggests a decrease in the efficiency of local neuronal activity, which ultimately leads to a failure of behavioral control (Bernardes et al., 2018). Gut flora analysis showed reduced abundance of Mycobacterium anisopliae in overweight/obese T2DM patients, which may weaken the inhibition of impulsive feeding by affecting prefrontal executive function (Dong et al., 2020). Notably, the left middle occipital gyrus and the thalamus, nucleus accumbens, amygdala, and other limbic systems showed enhanced FC in overweight/obese T2DM patients compared with those in the purely obese population, suggesting that over coupling between visual information processing and the reward system may drive the reinforcement of “food craving” behaviors (Eren-Yazicioglu et al., 2020; Wang et al., 2017), which is consistent with the findings of previous studies that T2DM patients with reward may be more susceptible to food cravings than those in the purely obese population. This is consistent with previous studies that enhanced functional connectivity of the reward network in T2DM patients may reflect increased sensitivity to high-calorie foods (Cazettes et al., 2011).

In terms of brain white matter structure, the present study found that AD values in the right lenticular nucleus were significantly lower in overweight/obese T2DM patients, and correlations existed with the feeding score, fruit preference score, and 24-h food intake, which may affect the cortico-striatal-thalamic loop to modulate ingestive behaviors. Previous researchers have similarly explored this issue in an obese group, and they found that fractional anisotropy in hypothalamic and hippocampal regions (FA) values correlated with the Shannon index in the alpha diversity index. In addition, these studies revealed a correlation between FA values in the amygdala and thalamus and the relative abundance of actinomycetes (Fernandez-Real et al., 2015).

Based on the results of gut flora analysis, we found that the increased abundance of *Prevotella* and *Bifidobacterium* may contribute to the conversion of tryptophan into kynurenine more often than 5-hydroxytryptophan (5-HT) by modulating the tryptophan metabolism pathway, and consequently leading to a decreased levels of 5-HT in the CNS, kynurenine can cross the blood–brain barrier to inhibit prefrontal cortex (PFC) function and impair inhibitory control behaviors (Oroojzadeh et al., 2022; Singh et al., 2022). *Prevotella* possesses the ability to dehydroxylate primary bile acids to generate secondary bile acids, such as deoxycholic acid, and other products, the latter of which exacerbate insulin resistance by inhibiting GLP-1 secretion via the farnesol X receptor (FXR) (Kong et al., 2019; Su et al., 2022). Elevation of *Bifidobacterium* may have a dual effect, on the one hand, inhibiting the NF-κB pathway and attenuating neuroinflammation through the secretion of metabolites such as indole-3-lactic acid, and on the other hand, data from animal experiments suggest that *Bifidobacterium* are able to promote the expression of tyrosine hydroxylase, which in turn elevates the synthesis of dopamine, which may enhance the rewarding effect of ingestion by augmenting dopaminergic signaling in the nucleus accumbens (Ji et al., 2025).

Meanwhile, reduced abundance of *Bacteroides*, *Fusobacterium*, and *Phascolarctobacterium* may decrease the production of short-chain fatty acids (SCFAs), further impairing the inhibitory effects of GLP-1 and PYY on hypothalamic feeding centers (Wei et al., 2021). *Fusobacterium* have the ability to ferment dietary fiber to produce propionic acid and butyric acid, which activates free fatty acid receptor 3 (FFAR3), which in turn stimulates the secretion of GLP-1 from enteroendocrine cells, suppresses appetite and enhances insulin sensitivity, and its reduced abundance may lead to a decrease in the level of SCFAs, weakening the inhibitory effect on the hypothalamic arched nucleus (ARC) of the AgRP/NPY neurons, thereby enhancing the desire to ingest (Wei et al., 2021). In addition, the reduction of *Phascolarctobacterium* may affect succinate metabolism, leading to mitochondrial dysfunction and increased oxidative stress, echoing the functional alterations in the abnormal ALFF region in the resting-state functional magnetic resonance index.

Finally, overweight/obese T2DM patients are often accompanied by chronic low-grade inflammation, and dysbiosis of the intestinal flora can lead to an increase in lipopolysaccharide (LPS), which activates peripheral and central immune responses. LPS promotes the release of proinflammatory factors through the Toll-like Receptor 4 (TLR4) signaling pathway, which induces hypothalamic inflammation and interferes with leptin signaling (Garg and Mohajeri, 2024). Enhanced FC values in the left middle occipital gyrus and amygdala isoforms in the present study may reflect the direct action of inflammatory factors on the limbic system to enhance emotional responses to food cues (Ji et al., 2025).

Based on the above mechanisms, therapeutic strategies can be explored in several directions: (1) dietary intervention and prebiotics: supplementation with prebiotics such as oligofructose and resistant starch selectively promotes the value-added of *Fusobacterium* and *Phascolarctobacterium* to increase the production of SCFAs and restore the GLP-1/PYY signaling pathway; (2) neuromodulation techniques: transcranial magnetic stimulation (TMS) or deep brain stimulation (DBS) targets the inhibition of the nucleus accumbens or enhance prefrontal cortex activity to balance the reward-inhibitory brain network; (3) hypoglycemic drugs and flora synergism: metformin in combination with probiotics synergistically improves insulin sensitivity and reduces kynurenine production by regulating tryptophan metabolism; (4) Personalized flora transplantation: targeted flora transplantation protocols are being developed to restore intestinal barrier function and metabolic homeostasis in individuals who are deficient in *Bacteroides* and *Fusobacterium*.

The gut-brain axis integrates energy metabolism and feeding behavior through neural, endocrine, and microbial signaling and is an important regulatory hub in obesity and diabetes. Although the results of this study demonstrated the complexity of the brain-flora association, there are still some limitations. This study is a cross-sectional design and has a small sample size, and the gut-flora-brain functional connectivity may change dynamically with the progression of the disease, so it is necessary to expand the sample size, and the longitudinal study combined with time-series analyses; second, the collection of dietary records and scales about the dietary records and scales was subjective and insufficiently standardized, which may affect the flora and ingestive behavior. In the future, it is necessary to combine macro-genomics, metabolomics and epigenetics data to deeply analyze the spatial and temporal distribution and signaling pathways of the gut-brain axis, so as to promote interdisciplinary therapeutic strategies for metabolic-neuropsychiatric disorders.

## 5 Conclusion

In this study, multimodal MRI and intestinal microbiology analysis revealed that, compared with the purely obese population, overweight/obese T2DM patients had significant alterations in brain structure, brain functional activity, and cerebral white matter in the prefrontal cortex, with enhancement of ALFF in the cerebellar hemispheres and right inferior frontal gyrus lid, enhancement of FC in the left middle occipital gyrus with the nucleus accumbens, amygdala, and lenticular nucleus, and significantly higher AD value of the DTI indicator in the lenticular nucleus decreased, and the relative abundance of *Bifidobacterium* and *Bacteroides* correlated with the indicators of altered brain function and appetite scores, which indicated that the abnormal desire to ingest in overweight/obese T2DM patients was a result of the joint action of functional remodeling of the brain area, dysregulation of the metabolism of the bacterial flora, and the interaction of the nerves and immunity. Through the integrated analysis of multimodal MRI and microbiomics, this study provides a theoretical basis for clinical diagnosis and treatment targeting the gut-brain axis, and the regulatory mechanisms of the gut-brain axis-specific network need to be further explored in the future.

## Data Availability

The datasets presented in this article are not readily available because patient privacy. Requests to access the datasets should be directed to 1207803073@qq.com.

## Footnotes

^1^ https://surfer.nmr.mgh.harvard.edu/fswiki/DownloadAndInstall

^2^ https://www.nitrc.org/projects/dcm2nii/

^3^ https://rfmri.org/DPABI

^4^ https://marsbar-toolbox.github.io/

^5^ http://dsi-studio.labsolver.org
